# Potent Chimeric Antimicrobial Derivatives of the *Medicago truncatula* NCR247 Symbiotic Peptide

**DOI:** 10.3389/fmicb.2020.00270

**Published:** 2020-02-21

**Authors:** Sándor Jenei, Hilda Tiricz, János Szolomájer, Edit Tímár, Éva Klement, Mohamad Anas Al Bouni, Rui M. Lima, Diána Kata, Mária Harmati, Krisztina Buzás, Imre Földesi, Gábor K. Tóth, Gabriella Endre, Éva Kondorosi

**Affiliations:** ^1^Institute of Plant Biology, Biological Research Centre, Szeged, Hungary; ^2^Department of Medical Chemistry, University of Szeged, Szeged, Hungary; ^3^Institute of Biochemistry, Biological Research Centre, Szeged, Hungary; ^4^Department of Laboratory Medicine, University of Szeged, Szeged, Hungary; ^5^Department of Oral Biology and Experimental Dental Research, University of Szeged, Szeged, Hungary; ^6^MTA-SZTE Biomimetic Systems Research Group, University of Szeged, Szeged, Hungary

**Keywords:** antimicrobial peptides, plant symbiotic nodule-specific cysteine-rich peptides, NCR247, ESKAPE bacteria, modes of antimicrobial activity, killing kinetics, bacterial targets, antibiotics

## Abstract

In Rhizobium-legume symbiosis, the bacteria are converted into nitrogen-fixing bacteroids. In many legume species, differentiation of the endosymbiotic bacteria is irreversible, culminating in definitive loss of their cell division ability. This terminal differentiation is mediated by plant peptides produced in the symbiotic cells. In *Medicago truncatula* more than ∼700 nodule-specific cysteine-rich (NCR) peptides are involved in this process. We have shown previously that NCR247 and NCR335 have strong antimicrobial activity on various pathogenic bacteria and identified interaction of NCR247 with many bacterial proteins, including FtsZ and several ribosomal proteins, which prevent bacterial cell division and protein synthesis. In this study we designed and synthetized various derivatives of NCR247, including shorter fragments and various chimeric derivatives. The antimicrobial activity of these peptides was tested on the ESKAPE bacteria; *Enterococcus faecalis*, *Staphylococcus aureus*, *Klebsiella pneumoniae*, *Acinetobacter baumannii*, *Pseudomonas aeruginosa*, and *Escherichia coli* as a member of *Enterobacteriaceae* and in addition *Listeria monocytogenes* and *Salmonella enterica*. The 12 amino acid long C-terminal half of NCR247, NCR247C partially retained the antimicrobial activity and preserved the multitarget interactions with partners of NCR247. Nevertheless NCR247C became ineffective on *S. aureus*, *P. aeruginosa*, and *L. monocytogenes*. The chimeric derivatives obtained by fusion of NCR247C with other peptide fragments and particularly with a truncated mastoparan sequence significantly increased bactericidal activity and altered the antimicrobial spectrum. The minimal bactericidal concentration of the most potent derivatives was 1.6 μM, which is remarkably lower than that of most classical antibiotics. The killing activity of the NCR247-based chimeric peptides was practically instant. Importantly, these peptides had no hemolytic activity or cytotoxicity on human cells. The properties of these NCR derivatives make them promising antimicrobials for clinical use.

## Introduction

The world-wide spread of antibiotic resistant bacteria and the increasing mortality rate by untreatable microbial infections make the development of new antibiotics with novel modes of actions and less prone to development of resistance extremely urgent. Plant peptides, produced only in Rhizobium bacterium-legume symbiosis, in the symbiotic cells of root nodules, represent a rich source of so far unexplored biological activities and antimicrobial agents. The infected nodule cells contain thousands of bacteria that are encapsulated by plasma membrane derived vesicles, forming organelle-like structures, called symbiosomes. In the host cells the bacteria adapt to the intracellular life-style, microaerobic conditions and differentiate progressively into nitrogen-fixing bacteroids. In many legumes, this differentiation process is irreversible, and manifested by extreme cell growth, altered morphology and physiology, genome amplifications and definitive loss of cell division potential (Kondorosi et al., 2013). We described this terminal differentiation process first in *Medicago truncatula* and demonstrated that it is controlled by the host plant (Mergaert et al., 2006). We discovered an entirely new peptide family which has evolved only in those legumes where the endosymbionts’ differentiation is terminal (Mergaert et al., 2003; Van de Velde et al., 2010). In *M. truncatula*, ∼700 genes code for nodule specific cysteine rich NCR peptides (Montiel et al., 2017; de Bang et al., 2017). The *NCR* genes usually consist of two exons; the first coding for a relatively conserved signal peptide, while the second one for the mature peptide. The mature NCR peptides exhibit extreme differences in their physicochemical properties due to their highly divergent amino acid compositions and sequence where only the position of 4 or 6 cysteines and a few neighboring amino acids is conserved. The NCR peptides enter the endoplasmic reticulum during their translation where the signal peptidase complex cleaves the signal peptide and the mature peptides reach the symbiosomes via the Golgi transport vesicles. NCR peptides can interact with the bacterial cell envelope, the bacterial membranes and with specific targets in the bacterial cytosol by entering the cells.

NCR247, which is the smallest peptide of the NCR family, is composed of 24 amino acids, four of which are cysteines. This cationic peptide (pI 10.15) is a self-penetrating peptide entering the bacterial cytosol without pore formation, which has exceptionally high protein binding ability and interacts with many bacterial proteins (Farkas et al., 2014). It binds to the conserved cell division protein FtsZ and abolishes the FtsZ ring formation and thereby bacterial cell division. NCR247 interacting with many ribosomal proteins inhibits translation but also downregulates the transcription of ribosomal genes. Accordingly, treatment of various pathogenic bacteria with synthetic NCR247 provoked efficient killing of many of them (Tiricz et al., 2013).

In this work, we studied the antimicrobial activity of NCR247 and its derivatives on the most problematic ESKAPE bacteria, *Enterococcus faecalis*, *Staphylococcus aureus*, *Klebsiella pneumoniae*, *Acinetobacter baumannii*, *Pseudomonas aeruginosa*, and *Escherichia coli* as well as *Listeria monocytogenes* and *Salmonella enterica*. We tested which region of NCR247 is responsible for the antibacterial activity and how this activity and antimicrobial spectrum can be further enhanced by fusion with neutral or other antimicrobial peptide (AMP) fragments. We show that the designed chimeric peptides act very fast and in at least as low or even lower concentrations than levofloxacin (Lvx) and orders of magnitude lower concentrations than carbenicillin (Cb). None of the five potent chimeric peptides provoked hemolysis of human erythrocytes or killed human cells, which makes them very promising antimicrobial candidates.

## Materials and Methods

### Bacterial Strains

The pathogenic bacterial strains obtained from the ATCC (United States) and NCTC (National Collection of Type Cultures – England) were the Gram-positive: *Enterococcus faecalis* (ATCC 29212), *Staphylococcus aureus* (HNCMO112011), *Listeria monocytogenes* (ATCC 19111) and the Gram-negative: *Pseudomonas aeruginosa* (ATCC 27853), *Escherichia coli* (ATCC 8739), *Salmonella enterica* (ATCC 13076), *Klebsiella pneumoniae* (NCTC 13440), *Acinetobacter baumannii* (ATCC 17978).

### Antimicrobial Activity in 20 mM Potassium-Phosphate Buffer, pH 7.4 (PPB)

Overnight bacterial cultures grown in LB were diluted and grown to OD_600_ = 0.2–0.6 at 37°C with shaking. After harvesting and washing bacteria in PPB, the suspensions were adjusted to OD_600_ = 0.1 corresponding to ∼10^7^ bacteria/mL. Antimicrobial activity of the peptides and the antibiotics was determined in PPB in 96 well microplates using 10 μL bacterial suspension and 90 μL of the 2-fold serial dilutions of peptides or antibiotics. Peptides were tested from 25 μM to 0.125 μM whereas the antibiotics; carbenicillin (Cb) from Duchefa Biochemie (Prod.No. C0109.0025) and levofloxacin (Lvx) from Sigma-Aldrich (Prod.No. 28266-10MG-F) from 10.24 mM to 0.1 μM. The microplates were incubated for 3 h at 37°C with 250 rpm shaking and then 5 μl from each sample was placed on LB agar and the growth of bacteria was monitored after overnight incubation at 37°C. The lowest concentration of the antimicrobial agents, which completely eliminated viable bacteria was the minimal bactericidal concentration (MBC).

### Antimicrobial Activity in Mueller Hinton Broth (MHB)

Ca^2+^ and Mg^2+^ can inhibit the antimicrobial activity of cationic peptides, therefore the MBC and MIC values were also determined in MHB, which unlike PPB contains divalent cations and allows growth of bacteria. Bacteria were grown and prepared similarly except that bacteria were resuspended in MHB. 10 μl of bacteria OD_600_ = 0.1, supplemented with 45 μl MHB and 45 μl of 2-fold dilution series of peptides, were cultivated for 3 and 20 h at 37°C with shaking. Optical density of each sample was measured (Hidex Sense Microplate Reader with Plate Reader Software version 5064) and 5 μl was placed on LB agar plates after 3 and 20 h treatment. The minimal inhibitory concentration (MIC) corresponded to the lowest concentration, which inhibited growth.

### Chemical Synthesis of Peptides

The antimicrobial peptides (AMPs) listed in Table 1 were synthesized according to the standard procedure of the solid-phase peptide synthesis (SPPS) by using an automatic peptide synthesizer (CEM Liberty Blue) with TentaGel S RAM resin (loading of amino groups −0.23 mmol/g). The applied chemistry utilized the Fmoc amino protecting group and diisopropylcarbodiimide/oxyma coupling with a fivefold excess of reagents. Removal of the fluorenyl-9-methoxycarbonyl (Fmoc) group was carried out with 10% piperazine and 0.1 mol 1-hydroxy-benzotriazole (HOBt) dissolved in 10% ethanol and 90% DMF in two cycles. After completion of the synthesis, peptides were detached from the resin with a 95:5 (v/v) trifluoroacetic acid (TFA)/water mixture containing 3% (w/v) dithiothreitol (DTT) and 3% (w/v) triisopropylsilane (TIS) at room temperature (RT) for 3 h. The resin was removed by filtration and the peptides were precipitated by the addition of ice cold diethyl ether. Next, the precipitate was filtered, dissolved in water and lyophilized. The crude peptides were analyzed and purified by reverse-phase high-performance liquid chromatography (RP-HPLC). Peptides were purified using a C18 column with a solvent system of (A) 0.1% (v/v) TFA in water and (B) 80% (v/v) acetonitrile and 0.1% TFA (v/v) in water at a flow rate of 4.0 mL/min. The absorbance was detected at 220 nm. The appropriate fractions were pooled and lyophilized. Purity of the peptides was characterized by analytical RP-HPLC at a flow rate of 1.0 mL/min. The identity of the peptides was verified by ESI-MS using Waters SQ detector.

**TABLE 1:** List of antimicrobial peptides.

**Code**	**Name**	**Amino acid sequence**	**pI**
**A**	NCR247	RNGCIVDPRCPY**QQCRRPLYCRRR**	10.15
**B**	**NCR247C**	**QQCRRPLYCRRR**	11.50
**C**	**NCR247C**-StrepII	**QQCRRPLYCRRR***WSHPQFEK*	11.05
**D**	X1-**NCR247C**	RPLNFKMLRFWGQ**QQCRRPLYCRRR**	11.99
**E**	X1-**NCR247C**-StrepII	RPLNFKMLRFWGQ**QQCRRPLYCRRR***WSHPQFEK*	11.80
**F**	**NCR247C**-X2	**QQCRRPLYCRRR**KALAALAKKIL	11.55
**G**	**NCR247C**-X2-StrepII	**QQCRRPLYCRRR**KALAALAKKIL*WSHPQFEK*	11.40
**H**	X2-**NCR247C**	KALAALAKKIL**QQCRRPLYCRRR**	11.65
**I**	X2	KALAALAKKIL	10.98
**J**	Transportan	GWTLNSAGYLLGKINLKALAALAKKIL	10.77

### Identification of the Interacting Bacterial Partners of NCR247C-X2-StrepII

Logarithmic phase *E. coli* bacteria (OD_600_ = 0.6) grown in LB were harvested by centrifugation, washed and re-suspended in PPB. Separation of the membrane and cytosolic proteins was done by ultracentrifugation according to Sandrini et al. (2014). Briefly, bacteria were sonicated and large debris removed by centrifugation. The membrane proteins (pellet) and the cytoplasmic proteins (supernatant) were separated by ultracentrifugation of the supernatant at 110000 *g* for 10 min at 4°C. NCR247C-X2-StrepII (peptide G, Table 1) or StrepII was added at 0.02 mM concentration to the membrane or cytosolic protein extract. These protein samples were incubated together with Strep-Tactin Sepharose beads (IBA Lifesciences Cat.No. 2-1201-010) for 90 min with gentle shaking on ice. Affinity chromatography of protein complexes was carried out according to the manual. Briefly, after the incubation, each column was washed seven times with washing buffer (100 mM Tris–HCl pH 8.0, 150 mM NaCl, 1 mM EDTA) and the bound proteins were eluted with elution buffer (100 mM Tris–HCl pH 8.0, 150 mM NaCl, 1 mM EDTA, 2.5 mM desthiobiotin). The 2nd and 3rd elution fractions (E2, E3) were separated with electrophoresis on 4–20% precast denaturing gel (Bio-Rad Laboratories Cat.No. 456-1093) and visualized with silver staining. Proteins were identified from E3 with mass spectrometry. Guanidine hydrochloride was added to the elution fractions to a final concentration of 6 M. The disulfide bridges were reduced by 10 mM Tris(2-carboxyethyl)phosphine (TCEP), then free sulfhydryls modified by 20 mM *S*-methyl methanethiosulfonate (MMTS). After dilution of the samples 0.5 μg trypsin was added for an overnight digestion at 37°C. The digests were desalted on Varian OMIX C18 tips and dried. An aliquot of the digests was subjected to LC-MS/MS analysis on an Orbitrap Fusion Lumos Tribrid (Thermo Fisher Scientific) mass spectrometer on-line coupled to a Waters M-Class UPLC using HCD fragmentation. The peak lists generated by Protein Discoverer (v1.4.) were subjected to database search using Protein Prospector (v5.22.0) against *E. coli* entries in the Uniprot.2019.6.12 database (1618419 entries).

### Hemolysis and Cytotoxicity Assays

To evaluate possible side effects of the NCR247-based peptides, hemolysis and cytotoxicity assays were performed. Human blood was purchased from the Regional Blood Centre in Szeged. The use of human blood for the hemolysis assay has been authorized by the Regional Hungarian Ethics Committee and approved by the Ethics Review Sector of DG RTD (European Commission) in connection with EK’s ERC AdG SymBiotics. The cells from 1.2 mL of EDTA-blood were centrifuged at 1500 × *g* for 1 min and washed several times in TBS buffer (10 mM Tris, pH = 7.2, 150 mM NaCl) until the supernatant became colorless. The cells were then resuspended in 12 mL TBS buffer. 100 μL of this cell suspension was incubated with 100 μL of the peptides at 100 and 25 μM. Samples were incubated for 1 h at 37°C, and after centrifugation of the cells at 1500 *g* for 1 min, the supernatants were transferred into sterile 96-well plates and the hemoglobin release was measured at OD_560_ (Multiskan FC microplate reader, Thermo Fisher Scientific). Melittin (Bachem) at 50 μg/mL and TBS without peptides were used as positive and negative controls, respectively. Relative hemolytic activity (RHA) of each peptide was calculated as follows: (Compound OD_560_ −TBS OD_560_) × 100/(Melittin OD_560_ −TBS OD_560_).

The cytotoxicity of peptides X1-NCR247C and NCR247C-X2 was determined against the human melanoma A375 cells with XTT cell proliferation assay kit (PanReac Applichem) and a benchtop microplate reader (Multiskan RC, Thermo Labsystems) according to the manufacturers’ instructions. A375 human melanoma cell line was obtained from ATCC and maintained in DMEM (Lonza) supplemented by 10% FBS (Euroclone) in a humidified incubator at 37°C and 5% CO_2_. A375 cells were seeded to 96-well plates (4,000 cells/well) and treated with the peptides for 72 h. Cells without peptides served as negative control and the blank sample was the medium. Reduction of the tetrazolium salt XTT by the viable cells to the orange colored compounds of formazan was measured at OD_450_. The XTT assay was performed in three biological repeats.

## Results

### NCR247 and Its Derivatives Are Potent Killers of Pathogenic Bacteria

Previously we have shown bactericidal activity of the synthetic NCR247 peptide in PBS on several human and plant pathogen bacteria (Tiricz et al., 2013). Our present work is focused on the activity of NCR247 and its various derivatives (Table 1) on the ESKAPE strains as well as on *L. monocytogenes* and *S. enterica*. Bacterial cultures were treated with 2-fold dilution series of synthetic NCR247 (peptide A, Table 1) starting with 25 μM concentration. The minimal bactericidal concentration (MBC) was the lowest concentration of the tested molecules where no viable bacteria remain after the treatment and therefore the growth of bacteria could not be detected (Table 2). The killing activity of NCR247 was the strongest with a MBC of 3.1 μM on *P. aeruginosa* while the MBC was 6.3 μM in the case of *S. aureus* and *E. coli*, 12.5 μM for *A. baumannii* and 25 μM for *S. enterica*. *K. pneumoniae*, and *L. monocytogenes* were resistant at 25 μM to NCR247 (peptide A, Table 2).

**TABLE 2:** Minimal bactericidal concentrations (MBC; in μM) of the studied peptides and antibiotics against different pathogens after 3 h of treatment in PPB.

**Peptides/Antibiotics**	***E. f.***	***S. a.***	***K. p.***	***A. b.***	***P. a.***	***E. c.***	***L. m.***	***S. e.***
**A**	>25	6.3	>25	12.5	3.1	6.3	>25	25
**B**	>25	>25	>25	25	25	6.3	>25	>25
**C**	>25	6.3	>25	25	6.3	6.3	>25	>25
**D**	6.3	3.1	12.5	3.1	3.1	3.1	3.1	1.6
**E**	12.5	3.1	6.3	3.1	3.1	3.1	3.1	3.1
**F**	25	3.1	6.3	3.1	3.1	1.6	1.6	1.6
**G**	12.5	1.6	3.1	1.6	3.1	1.6	3.1	1.6
**H**	3.1	3.1	6.3	3.1	3.1	3.1	3.1	3.1
**I**	>25	25	>25	25	6.3	>25	25	>25
**J**	3.1	3.1	3.1	1.6	3.1	3.1	3.1	1.6
**Cb**	5120	640	>10240	5120	10240	1280	80	640
**Lvx**	160	2.5	320	20	1.3	5.0	320	1.3

**E. f.*, *Enterococcus faecalis*; *S. a*., *Staphylococcus aureus*; *K. p.*, *Klebsiella pneumoniae*; *A. b.*, *Acinetobacter baumannii*; *P. a*., *Pseudomonas aeruginosa*; *E. c.*, *Escherichia coli*; *L. m.*, *Listeria monocytogenes*; *S. e.*, *Salmonella enterica*. Cb, carbenicillin; Lvx, levofloxacin.*

Then we tested which region of NCR247 is responsible for the antimicrobial properties. From the 24 amino acid long NCR247, the 12 amino acid long N-terminal and the 12 amino acid long C-terminal halves were synthetized. The N-terminal part (RNGCIVDPRCPY) was inactive on the tested bacteria, while the C-terminal part (peptide B: NCR247C, Table 1) retained the antimicrobial activity of NCR247 on *E. coli* (peptide B, Table 2) but was ineffective to kill the other bacteria at 25 μM concentration.

The synthetic, reduced and even the oxidized forms of NCR247 seems to be unstructured (Shabab et al., 2016). However, it cannot be excluded that NCR247 might be properly folded by interacting with the bacterial membranes, which may not be possible if the peptide is only 12 amino acid long. Therefore, we synthetized derivatives of the NCR247C peptide adding either C- or N-terminal extension. First, the neutral, 8 amino acid long StrepII tag (WSHPQFEK) was added to the C terminus (peptide C: NCR247C-StrepII, Table 1). The StrepII was chosen because in our previous study this tag did not harm the antimicrobial activity of NCR247 and it was used for pull down experiments to identify its bacterial targets (Farkas et al., 2014). NCR247C-StrepII became more active than NCR247C killing in addition to *E. coli*, *S. aureus*, and *P. aeruginosa* (peptide C, Table 2).

Then the N-terminus of NCR247C was extended with 13 amino acids (X1) deriving from the cationic N-terminal part of the unusual double size NCR335 which part lacks cysteines and characteristics of NCRs but increased the pI from 10.15 to 11.99 (peptide D, Table 1). This chimeric peptide (X1-NCR247C) turned out to be very effective on *S. enterica* (MBC 1.6 μM), *S. aureus*, *A. baumannii*, *P. aeruginosa*, *E. coli*, and *L. monocytogenes* (MBC 3.1 μM) and became able to kill *E. faecalis* (MBC 6.3 μM) and *K. pneumoniae* (MBC 12.5 μM) (peptide D, Table 2). Addition of StrepII to the C terminus of X1-NCR247C (peptide E: X1-NCR247C-StrepII, Table 1) slightly improved the bactericidal property against *K. pneumoniae* but became somewhat less efficient against *E. faecalis* and *S. enterica* (peptide E, Table 2).

Thereupon, we investigated how attachment of another AMP at the C- or N-terminus of NCR247C could influence the bactericidal efficiency and spectrum. We used the KALAALAKKIL sequence (peptide I: X2, Table 1) from the membranolytic, anti-cancer mastoparan peptide toxin from wasp venom (INLKALAALAKKIL), which is also present in the C-terminus of the 27 amino acid long cell-penetrating cationic peptide, transportan (peptide J: Transportan, Table 1) (Pooga et al., 1998). Attachment of KALAALAKKIL to the C-terminus of NCR247C (peptide F: NCR247C-X2, Table 1) reduced the MBC to 1.6 μM in the case of *E. coli*, *L. monocytogenes*, and *S. enterica* (peptide F, Table 2). Further elongation of NCR247C-X2 with StrepII (peptide G: NCR247C-X2-StrepII, Table 1) made this derivative even more effective against *S. aureus* and *A. baumannii* (MBC 1.6 μM) (peptide G, Table 2). Addition of X2 to the N-terminal of NCR247C (peptide H: X2-NCR247C, Table 1) drastically increased the killing of *E. faecalis* and was the most active one (MBC 3.1 μM) among all tested peptide derivatives (peptide H, Table 2). X2 alone was incomparably less active on all tested bacteria (peptide I, Table 2). On the other hand transportan effectively killed all bacteria with MIC in the range of 1.6–3.1 μM (peptide J, Table 2).

### The Antimicrobial Potential of NCR247 Based Peptides Is Comparable to Third-Generation Antibiotics

To evaluate the effectiveness of these peptides compared to antibiotics, we determined the MBC values of two antibiotics under the same experimental conditions, after 3 h treatments in PPB. One of them was the classical bactericidal antibiotic carbenicillin, belonging to the carboxypenicillin subgroup of the penicillins. The other one was levofloxacin, a third-generation, fluoroquinolone antibiotic. The MBCs of both antibiotics showed great variations on the tested bacteria (Table 2). Carbenicillin, except for *L. monocytogenes*, was required in the range of 640 – 10240 μM while levofloxacin was active at 1.25 – 320 μM, comparable to the chimeric NCR247C derivatives. Levofloxacin was particularly effective against *P. aeruginosa*, *S. enterica*, and *S. aureus* but the MBCs against *K. pneumoniae*, *L. monocytogenes*, and *E. faecalis* were one or two orders of magnitude higher than that of the chimeric NCR peptides.

**FIGURE 1:**
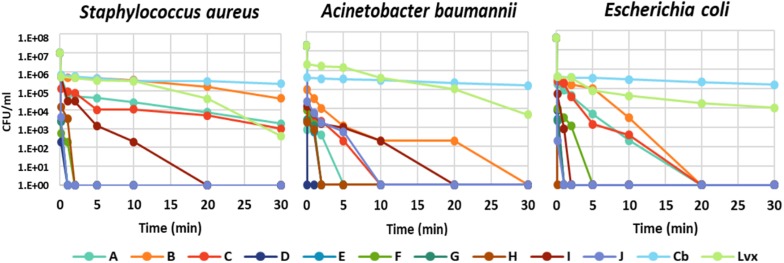
Time-kill kinetic analysis of antibiotics and the designed peptides. The number of colony forming units (CFU) is shown at 0.0, 0.1, 1, 2, 5, 10, 20, and 30 min of treatment.

The time dependence of bacterial killing was a further question, which act faster: the peptides or the antibiotics? The kinetics of bacterial killing was studied on *S. aureus*, *A. baumannii*, and *E. coli* treated with MBC of each antimicrobial molecule (peptide or antibiotic) (Figure 1 and Supplementary Table S1). All chimeric peptides (D-H) killed all tested bacteria either instantly or within 5 min. The killing by NCR247 and its short derivatives (A-C) was slower but they eliminated *E. coli* in 20 min and *A. baumannii* in 30 min while viable cell number of *S. aureus* was only slightly reduced in line with insensitivity of *S. aureus* to the A–C peptides. Carbenicillin and levofloxacin exhibited a much more protracted killing effect than the NCR247-based peptides with large numbers of viable cells at 60 min (Supplementary Table S1). Based on this data, it is clear that particularly the chimeric peptides (D-H) kill bacteria much faster than the tested antibiotics.

### Bactericidal Activity of the Chimeric NCR247 Based Peptides Is Maintained in Mueller Hinton Broth

Antimicrobial activity of cationic peptides is generally attenuated by the presence of divalent cations and higher salt concentrations (Hancock and Sahl, 2006). Therefore we determined the MBC and the minimal inhibitory concentration (MIC) values of all peptides (A-J) in MHB used for the cultivation of *S. aureus*, *A. baumannii*, and *E. coli* (Table 3). Peptides A-C, corresponding to NCR247, NCR247C and NCR247C-StrepII were ineffective against the three bacteria cultivated for 3 or 20 h at 25 μM concentration. Growth inhibition was only detectable in the case of A: NCR247 at 25 μM on *E. coli* cultivated for 20 h. Unlike A-C, the D-H peptides retained their activity in MHB. Action of D and E was, however, slowed down, as their MBC values were significantly higher at 3 h than at 20 h. In contrast, the MBC values of peptides F, G and H were identical or almost the same at 3 and 20 h. Moreover in line with fast action of these peptides, the MIC and MBC values were practically identical.

**TABLE 3:** Minimal bactericidal concentrations (MBC) and minimal inhibitory concentrations (MIC) of the studied peptides in Mueller Hinton Broth on selected pathogens.

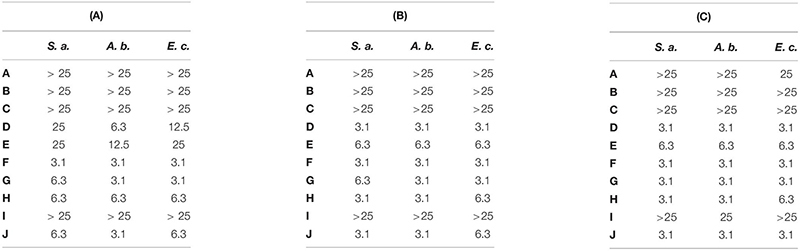

***(A)** MBC (μM) – 3 h treatment, **(B)** MBC (μM) – 20 h treatment, and **(C)** MIC (μM) – 20 h treatment. *S. a.*, *Staphylococcus aureus*; *A. b.*, *Acinetobacter baumannii*; *E. c.*, *Escherichia coli*.*

### The Chimeric Peptide G: NCR247C-X2-StrepII Interacts With Many *E. coli* Proteins

**FIGURE 2:**
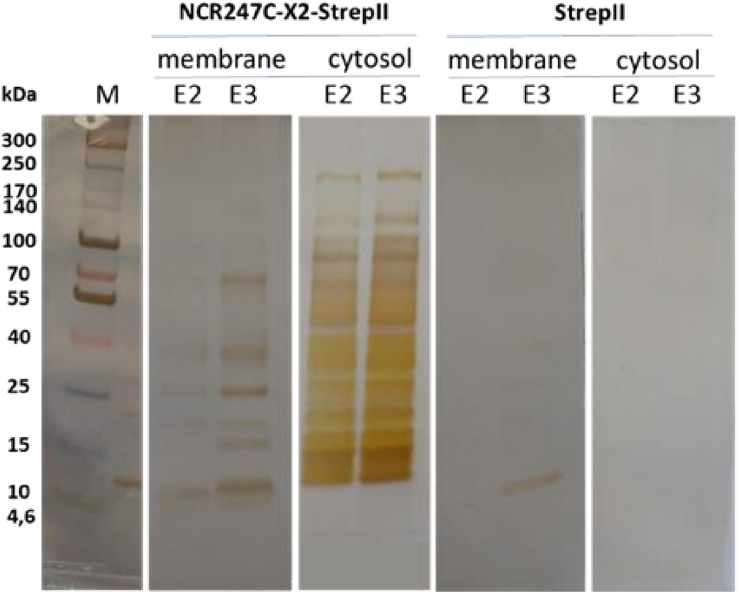
SDS-gel electrophoresis of proteins eluted from Strep-Tactin Sepharose column. E2, E3: 2nd and 3rd elution fractions. M: molecular weight markers.

**FIGURE 3:**
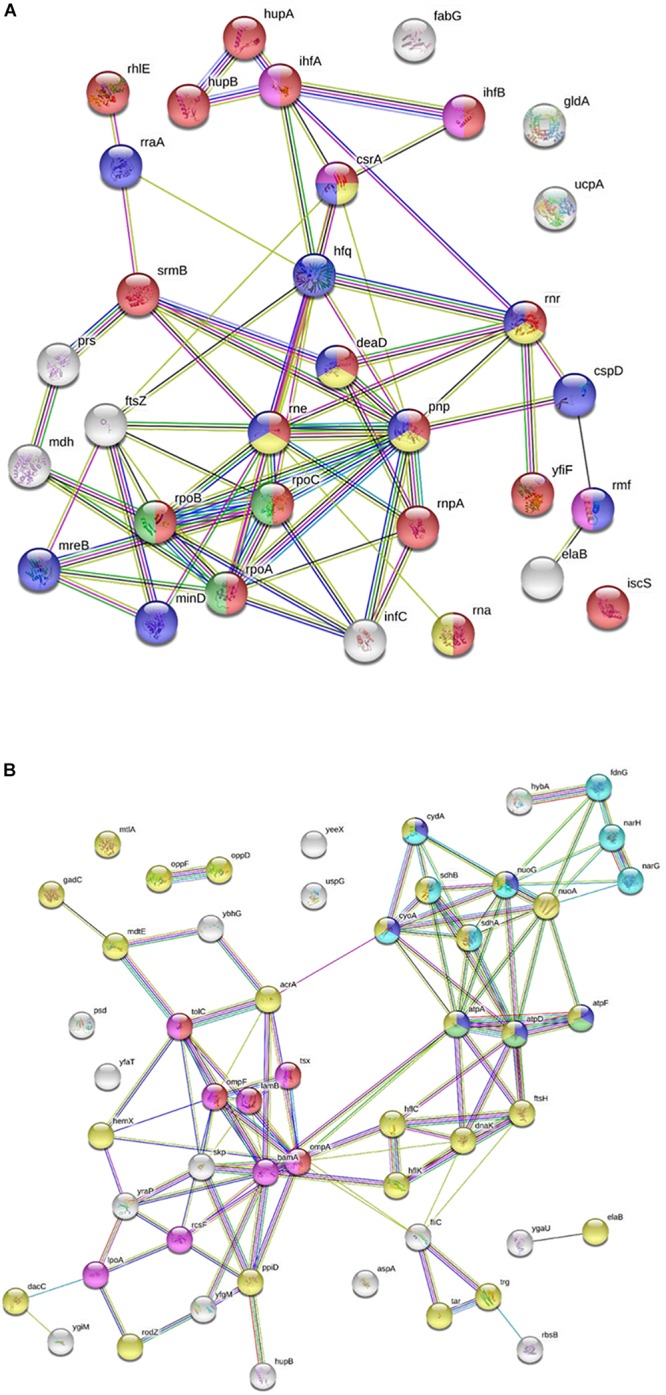
Interaction of NCR247C-X2-StrepII with *Escherichia coli* cytosolic and membrane proteins. **(A)** STRING representation of the cytosolic interacting proteins excluding the ribosomal proteins and other proteins involved in translation. The color of nodes indicates the following functions: red, RNA metabolic process; yellow, RNA catabolic process; blue, negative regulators; green, RNA polymerase; purple, translation regulation. The connecting lines indicate the interactions. **(B)** STRING representation of the interacting membrane proteins. Protein functions are indicated with different colors. The color of nodes indicate different functions: blue, ATP metabolic process; magenta, electron transport chain; red, porin activity; green, proton transport; yellow, inner membrane component and purple, outer membrane components.

Peptide G was one of the most active chimeric peptides, which via its StrepII tag attached to the C-terminus of NCR247C-X2, was used in pull-down experiments for the isolation of the interacting bacterial proteins. Cytosolic and membrane fractions of *E. coli* were incubated with NCR247C-X2-StrepII or with StrepII, serving as a control for identification of proteins binding to the StrepII tag. The protein complexes were purified with affinity chromatography using Strep-Tactin Sepharose beads and the eluted proteins were identified with mass spectrometry (MS) (Supplementary Table S2) and visualized by gel electrophoresis and silver staining (Figure 2). Numerous cytosolic proteins showed interaction with NCR247C-X2. They were predominantly ribosomal proteins that corresponded to the full list of ribosomal proteins identified previously as binding partners of NCR247-StrepII in the symbiotic partner *Sinorhizobium meliloti* (Farkas et al., 2014). These proteins ordered from the highest number of peptide counts by the MS analysis were the followings: L15, S5, S7, L4, L2, L9, L17, S4, L1, L3, L22, S9, S6, S13, S14, S3, L16, S19, L18, L13, L10, S18, S12, S28 (Supplementary Table S2). In addition, factors involved in ribosome maturation or RNA degradation (DeaD, Rne, Pnp, Hfq, Rnr, RhlE), in translation (InfC), in transcriptional and translational control (HupB, IhfA, IhfB, CsrA, RmF) including the RNA polymerase subunits (RpoA, RpoB, RpoC) were present in the NCR247C-X2-StrepII complexes (Figure 3A). FtsZ, a major binding partner of NCR247 and essential for the formation of the Z-ring and cell division, was represented with low peptide counts in the cytosolic fraction, indicating that the N-terminal half of NCR247 might contribute to the strong FtsZ binding. Similarly to FtsZ, MinD septum site-determining protein was detected with low peptide counts (Figure 3A).

There were fewer interacting partners in the membrane fraction (Supplementary Table S2) that were outer membrane components (LpoA, OmpA, OmpF, OmpW, RodZ, DacC, RcsF, BamA), inner membrane components (ElaB, AtpA, AtpD, AtpF, HemX, RodZ, FtsH, NuoA) and various efflux pumps (AcrA-TolC-MacA, MdtE) pumping out the toxic substances or antibiotics (Figure 3B).

### The NCR247-Based Peptides Have No Cytotoxicity Against Human Cells

Many antibiotics have severe side effects and cytotoxicity; therefore it was important to test how these plant peptides affect the integrity and viability of human cells. One of the simplest and essential assays is to measure hemolysis of erythrocytes. Hemolysis of human erythrocytes by A–J at 100 and 25 μM concentrations was measured and compared to the effect of melittin, an AMP from honey bee venom that induces complete hemolysis at 50 μg/mL. Blood cells without AMPs in TBS buffer served as negative control (Table 4). Only transportan showed hemolytic activity, which was 95.6 and 38.6% of the melittin provoked hemolysis at 100 and 25 μM, respectively. Interestingly, the truncated form (I) of the membranolytic mastoparan lost its hemolytic activity. Only the chimeric peptides F-H provoked negligible hemolysis at 100 μM. At 25 μM only peptide G had a slight and insignificant hemolytic activity.

**TABLE 4:** NCR247 and its derivatives do not provoke hemolysis.

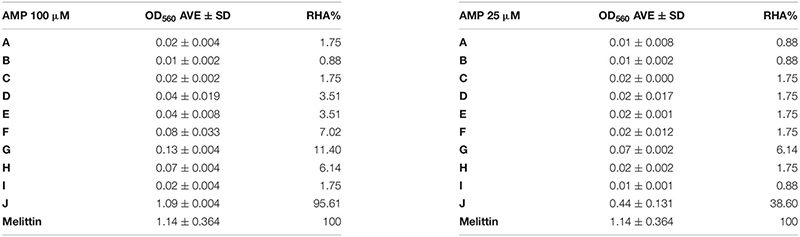

*Desintegration of red blood cells treated with 100 and 25 μM of AMPs A-J and melittin (50 μg/ml) was measured at OD_560_. Average OD_560_ ± standard deviation (OD_560_AVE ± SD) and the relative hemolytic activities (RHA) are presented in % of the 100% hemolytic effect of melittin.*

**FIGURE 4:**
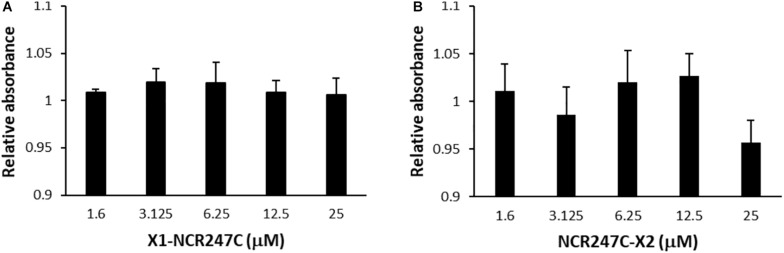
Viability of human melanoma cell line A375 treated with **(A)** X1-NCR247C or **(B)** NCR247C-X2. Relative absorbance values represent the viability compared to untreated cells. Bar graphs represent mean ± SD values.

In addition we tested the cytotoxicity of peptides X1-NCR247C and NCR247C-X2 on the viability of A375 human melanoma cells using the XTT cell proliferation assay (Figure 4). Conversion of XTT to formazan by metabolically active cells did not show differences between the control and the X1-NCR247C treated samples. Similarly NCR247C-X2 exhibited no cytotoxicity up to 12.5 μM, though slight decrease of the viable cell number was observed at 25 μM, which concentration is significantly higher than the MBC values. These data indicate that X1-NCR247C and NCR247C-X2 in the range of MBCs do not affect the viability and proliferation of this cell culture.

## Discussion

Based on the definitive loss of cell division ability of the nitrogen fixing bacteroids in *M. truncatula* root nodules, it is expected that at least a fraction of the ∼700 NCR peptides can inhibit bacterial cell division and act as antimicrobial agents *in vitro*. Indeed, in our previous studies we have shown the bactericidal action of several cationic NCR peptides (Van de Velde et al., 2010; Tiricz et al., 2013; Farkas et al., 2014; Mikuláss et al., 2016; Farkas et al., 2017; Farkas et al., 2018). Among them, the mode of action is the best known for NCR247 (Haag et al., 2012; Farkas et al., 2014; Penterman et al., 2014; Arnold et al., 2017). This peptide has numerous advantageous properties, it acts very fast, it has extreme protein binding ability and has many bacterial targets in the cytosol, by binding to FtsZ it abolishes bacterial cell division and by interacting with many ribosomal proteins it inhibits protein synthesis. Due to its multi hit mechanism, the probability of development of resistant bacteria against NCR247 is very low. A further advantage is that NCR247 is not cytotoxic for human cells.

In this work we show that NCR247 is capable of killing several, but not all of the most problematic bacteria causing incurable infections. Therefore, we aimed to further improve its bactericidal properties. Testing the antimicrobial activity of the synthetic N-terminal and C-terminal halves of NCR247 revealed that antimicrobial activity resided mostly in the C-terminal region (NCR247C). While the positive charge of the AMPs is essential for killing, in the case of NCR247C the increased positive charge (pI = 11.50) did not result in the improvement of the antimicrobial activity or the antimicrobial spectrum compared to NCR247 (pI = 10.15). NCR247C had even a much weaker antimicrobial activity which may be due to the lack of its proper folding as a result of its short length and/or to its failure to interact with the bacterial membranes. Adding the neutral StrepII tag to the NCR247C peptide improved slightly the antibacterial properties. Extending the N-terminus of NCR247C with the 13 amino acid long cationic peptide from NCR335 (D) increased the pI to 11.99 and resulted in significantly lower MBCs (1.6–12.5 μM) and broader spectrum; killing all the eight bacterium strains. Addition of the truncated mastoparan (lacking the first three N-terminal amino acids) either to the N- or the C-terminus of the NCR247C (F, H) increased in similar extent the antimicrobial properties. Further prolongation of the chimeric peptides with the StrepII tag (G) increased even more the activity reaching 1.6–3.1 μM of MBC on all bacteria except for *E. faecalis* (MBC 12.5 μM). The killing by the chimeric peptides was instant (approx. 0.1 min) or very fast (max 5 min) while the complete killing by the levofloxacin required more than 60 min and the carbenicillin acted even slower. In addition to the antibiotics’ slower killing kinetics, very high doses (up to 10240 μM) were required from carbenicillin for the elimination of the tested bacteria. The MBC values of levofloxacin was more comparable to chimeric peptides (D-H), acting on *S. aureus*, *A. baumannii*, *P. aeruginosa*, *E. coli*, and *S. enterica* in the same concentration range but being somewhat less active on *K. pneumoniae* and *L. monocytogenes* and *E. faecalis*. Importantly the chimeric peptides maintained their activity also in Mueller Hinton Broth where cationic AMPs are usually inactivated by the presence of bivalent cations (Ca^2+^, Mg^2+^), which prevent their interaction with the bacterial membranes (Hancock and Sahl, 2006).

In the chimeric peptide G, the advantageous properties of NCR247C and mastoparan were combined and the StrepII tag in addition made possible the identification of the interacting partners in *E. coli* using pull-down experiments coupled to mass spectrometry. NCR247 showed strong interaction with many ribosomal proteins, FtsZ and GroEL of the symbiotic bacterium partner *Sinorhizobium meliloti* (Farkas et al., 2014). The hybrid G peptide interacted with many *E. coli* proteins. Ribosomal proteins represented the large majority of cytosolic partners in agreement with the list of ribosomal proteins interacting with NCR247 and confirmed that NCR247C is responsible for binding of ribosomal proteins. Even if NCR247 has an extreme protein binding ability, it is unlikely that all these proteins are primary partners. Nevertheless, interaction with a few ribosomal proteins can be sufficient to inhibit translation and prevent the development of resistance. Already several ribosome-targeting peptide antibiotics are known which interact with different subunits, various ribosomal proteins and can effect initiation, elongation cycle, termination and recycling during protein synthesis (Polikanov et al., 2018). However, none of these peptides have such a broad interaction with the translation machinery as the NCR247-based peptides.

FtsZ was detected among the binding partners of peptide G, however, FtsZ was identified with lower peptide counts; nevertheless the interaction with FtsZ and MinD might collectively contribute to abolishment of bacterial cell division. Interestingly, GroEL playing essential roles in symbiosis was not found among the *E. coli* binding partners.

Mastoparan is a membranolytic 14-residue peptide toxin, which interacts with membranes and adopts amphipathic α-helical structure in lipid environment, capable of penetrating biological membranes and causing permeabilization of both bacterial and mammalian membranes culminating in cell lysis (Moreno and Giralt, 2015; Irazazabal et al., 2016). Unexpectedly, by the loss of the first three amino acids, the truncated mastoparan (X2) did not provoke hemolysis but accordingly lost much of its antimicrobial activity. Fusing X2 with NCR247C provoked synergistic effect, greatly improving the antimicrobial efficacy. The pull down with NCR247C-X2-StrepII identified several outer membrane and inner membrane components that probably bind to the X2 region of the chimeric peptide. Among them are various efflux pumps, pumping out the toxic substances or antibiotics (Amaral et al., 2014). Attachment of X1 to NCR247C enhanced similarly the antimicrobial properties.

In summary, we successfully designed and generated from the symbiotic antimicrobial plant peptide NCR247 several chimeric peptides with fast killing action, low MBC values, and numerous bacterial targets without toxicity on human cells. These peptides represent an extremely promising novel generation of highly powerful antimicrobial candidates.

## Data Availability Statement

All datasets generated for this study are included in the article/Supplementary Material.

## Author Contributions

Antimicrobial tests were done by SJ, ET, MA, and RL. HT and ÉKl performed the pull down experiments and identification of bacterial interacting proteins. Hemolytic assays were done by DK and IF and the cytotoxicity test by MH and KB. Peptides were designed by GT, ÉKo, and GE, and synthesized by JS and GT. Experiments were designed and evaluated by ÉKo, GE, SJ, and GT. ÉKo wrote the manuscript.

## Conflict of Interest

The authors declare that the research was conducted in the absence of any commercial or financial relationships that could be construed as a potential conflict of interest.

## Supplementary Material

TABLE S1 Killing kinetics of the studied AMPs and antibiotics at their respective MBC. Figure 1 is based on these data from 0 to 30 min.

TABLE S2 Interacting partners of NCR247C-X2-StrepII and the control StrepII peptide identified in the cytosolic and membrane fractions of *Escherichia coli*.

## References

[B1] Amaral L. Martins A. Spengler G. Molnar J. (2014). Efflux pumps of Gram-negative bacteria: what they do, how they do it, with what and how to deal with. *Front. Pharmacol.* 4:168. 10.3389/fphar.2013.00168 24427138 PMC3879458

[B2] Arnold M. F. F. Shabab M. Penterman J. Boehme K. L. Griffitts J. S. Walker G. C. (2017). Genome-wide sensitivity analysis of the microsymbiont Sinorhizobium meliloti to symbiotically important, defensin-like host peptides. *mBio* 8 e1060–e1017. 10.1128/mBio.01060-17 28765224 PMC5539429

[B3] de Bang T. C. Lundquist P. K. Dai X. Boschiero C. Zhuang Z. Pant P. (2017). Genome-wide identification of medicago peptides involved in macronutrient responses and nodulation. *Plant. Physiol.* 175 1669–1689. 10.1104/pp.17.01096 29030416 PMC5717731

[B4] Farkas A. Maróti G. Durgo H. Györgypál Z. Lima R. M. Medzihradszky K. F. (2014). *Medicago truncatula* symbiotic peptide NCR247 contributes to bacteroid differentiation through multiple mechanisms. *Proc. Natl. Acad. Sci. U.S.A.* 111 5183–5188. 10.1073/pnas.1404169111 24706863 PMC3986156

[B5] Farkas A. Maróti G. Kereszt A. Kondorosi E. (2017). Comparative analysis of the bacterial membrane disruption effect of two natural plant antimicrobial peptides. *Front. Microbiol.* 8:51. 10.3389/fmicb.2017.00051 28167938 PMC5253368

[B6] Farkas A. Pap B. Kondorosi E. Maróti G. (2018). Antimicrobial activity of NCR plant peptides strongly depends on the test assays. *Front. Microbiol.* 9:2600. 10.3389/fmicb.2018.02600 30425705 PMC6218624

[B7] Haag A. F. Kerscher B. Dall’Angelo S. Sani M. Longhi R. Baloban M. (2012). Role of cysteine residues and disulfide bonds in the activity of a legume root nodule-specific, cysteine-rich peptide. *J. Biol. Chem.* 287 10791–10798. 10.1074/jbc.M111.311316 22351783 PMC3322895

[B8] Hancock R. E. W. Sahl H. G. (2006). Antimicrobial and host-defense peptides as new anti-infective therapeutic strategies. *Nat. Biotechnol.* 24 1551–1557. 10.1038/nbt1267 17160061

[B9] Irazazabal L. N. Porto W. F. Ribeiro S. M. Casale S. Humblot V. Ladram A. (2016). Selective amino acid substitution reduces cytotoxicity of the antimicrobial peptide mastoparan. *Biochim. Biophys. Acta.* 1858 2699–2708. 10.1016/j.bbamem.2016.07.001 27423268

[B10] Kondorosi E. Mergaert P. Kereszt A. (2013). A paradigm for endosymbiotic life: cell differentiation of *Rhizobium* bacteria provoked by host plant factors. *Annu. Rev. Microbiol.* 67 611–628. 10.1146/annurev-micro-092412-155630 24024639

[B11] Mergaert P. Nikovics K. Kelemen Z. Maunoury N. Vaubert D. Kondorosi A. (2003). A novel family in *Medicago truncatula* consisting of more than 300 nodule-specific genes coding for small, secreted polypeptides with conserved cysteine motifs. *Plant Physiol.* 132 161–173. 10.1104/pp.102.018192 12746522 PMC166962

[B12] Mergaert P. Uchiumi T. Alunni B. Evanno G. Cheron A. Catrice O. (2006). Eukaryotic control on bacterial cell cycle and differentiation in the Rhizobium-legume symbiosis. *Proc. Natl. Acad. Sci. U.S.A.* 103 5230–5235. 10.1073/pnas.0600912103 16547129 PMC1458823

[B13] Mikuláss K. R. Nagy K. Bogos B. Szegletes Z. Kovács E. Farkas A. (2016). Antimicrobial nodule-specific cysteine-rich peptides disturb the integrity of bacterial outer and inner membranes and cause loss of membrane potential. *Ann. Clin. Microbiol. Antimicrob.* 15:43. 10.1186/s12941-016-0159-8 27465344 PMC4964015

[B14] Montiel J. Downie J. A. Farkas A. Bihari P. Herczeg R. Bálint B. (2017). Morphotype of bacteroids in different legumes correlates with the number and type of symbiotic NCR peptides. *Proc. Natl. Acad. Sci. U.S.A.* 114 5041–5046. 10.1073/pnas.1704217114 28438996 PMC5441718

[B15] Moreno M. Giralt E. (2015). Three valuable peptides from bee and wasp venoms for therapeutic and biotechnological use: melittin, apamin and mastoparan. *Toxins* 7 1126–1150. 10.3390/toxins7041126 25835385 PMC4417959

[B16] Penterman J. Abo R. P. De Nisco N. J. Arnold M. F. Longhi R. Zanda M. (2014). Host plant peptides elicit a transcriptional response to control the *Sinorhizobium meliloti* cell cycle during symbiosis. *Proc. Natl. Acad. Sci. U.S.A.* 111 3561–3566. 10.1073/pnas.1400450111 24501120 PMC3948309

[B17] Polikanov Y. S. Aleksashin N. A. Beckert B. Wilson D. N. (2018). The mechanisms of action of ribosome-targeting peptide antibiotics. *Front. Mol. Biosci.* 5:48. 10.3389/fmolb.2018.00048 29868608 PMC5960728

[B18] Pooga M. Hällbrink M. Zorko M. Langel U. (1998). Cell penetration by transportan. *FASEB J.* 12 67–77. 10.1096/fasebj.12.1.67 9438412

[B19] Sandrini S. M. Haigh R. Freestone P. P. P. (2014). Fractionation by ultracentrifugation of gram negative cytoplasmic and membrane proteins. *Bio Protocol.* 4:e1287 10.21769/BioProtoc.1287

[B20] Shabab M. Arnold M. F. Penterman J. Wommack A. J. Bocker H. T. Price P. A. (2016). Disulfide cross-linking influences symbiotic activities of nodule peptide NCR247. *Proc. Natl. Acad. Sci. U. S. A.* 113 10157–10162. 10.1073/pnas.1610724113 27551097 PMC5018749

[B21] Tiricz H. Szucs A. Farkas A. Pap B. Lima R. M. Maróti G. (2013). Antimicrobial nodule-specific cysteine-rich peptides induce membrane depolarization-associated changes in the transcriptome of *Sinorhizobium meliloti*. *Appl. Environ. Microbiol.* 79 6737–6746. 10.1128/AEM.01791-13 23995935 PMC3811505

[B22] Van de Velde W. Zehirov G. Szatmari A. Debreczeny M. Ishihara H. Kevei Z. (2010). Plant peptides govern terminal differentiation of bacteria in symbiosis. *Science* 327 1122–1126. 10.1126/science.1184057 20185722

