# Supplementary material for: Potent Chimeric Antimicrobial Derivatives of the Medicago truncatula NCR247 Symbiotic Peptide
Source: Front Microbiol. 2020 Feb 21;11:270. doi: 10.3389/fmicb.2020.00270 (PMC7047876; doi:10.3389/fmicb.2020.00270)
Supplement: TABLE S1 — Killing kinetics of the studied AMPs and antibiotics at their respective MBC. Figure 1 is based on these data from 0 to 30 min. [file Data_Sheet_1.DOCX]

**Supplementary Table S1**

Killing kinetics of the studied AMPs and antibiotics at their respective MBC. Figure 1 is based on these data from 0 to 30 min.

| ***Staphylococcus aureus*** | | | | |  |  |  |  |  |  |  |  |
| --- | --- | --- | --- | --- | --- | --- | --- | --- | --- | --- | --- | --- |
| **Time (min)** | **A** | **B** | **C** | **D** | **E** | **F** | **G** | **H** | **I** | **J** | **Cb** | **Lvx** |
| **0** | 1E+07 | 1E+07 | 1E+07 | 1E+07 | 1E+07 | 1E+07 | 1E+07 | 1E+07 | 1E+07 | 1E+07 | 1E+07 | 1E+07 |
| **0.1** | 6E+05 | 6E+05 | 1E+05 | 200 | 2600 | 540 | 2400 | 14000 | 6E+05 | 4000 | 7E+05 | 6E+05 |
| **1** | 64000 | 5E+05 | 90600 | 0 | 0 | 200 | 0 | 3400 | 28600 | 0 | ND | ND |
| **2** | 52400 | 5E+05 | 78200 | 0 | 0 | 0 | 0 | 0 | 28000 | 0 | 6E+05 | 5E+05 |
| **5** | 40800 | 5E+05 | 10200 | 0 | 0 | 0 | 0 | 0 | 1400 | 0 | 5E+05 | 4E+05 |
| **10** | 24600 | 4E+05 | 10400 | 0 | 0 | 0 | 0 | 0 | 200 | 0 | 3E+05 | 3E+05 |
| **20** | 7400 | 2E+05 | 5000 | 0 | 0 | 0 | 0 | 0 | 0 | 0 | 3E+05 | 37400 |
| **30** | 1800 | 39600 | 1000 | 0 | 0 | 0 | 0 | 0 | 0 | 0 | 2E+05 | 400 |
| **60** | ND | ND | ND | ND | ND | ND | ND | ND | ND | ND | 53400 | 0 |

| ***Acinetobacter baumannii*** | | | | |  |  |  |  |  |  |  |  |
| --- | --- | --- | --- | --- | --- | --- | --- | --- | --- | --- | --- | --- |
| **Time (min)** | **A** | **B** | **C** | **D** | **E** | **F** | **G** | **H** | **I** | **J** | **Cb** | **Lvx** |
| **0** | 2E+07 | 2E+07 | 2E+07 | 2E+07 | 2E+07 | 2E+07 | 2E+07 | 2E+07 | 2E+07 | 2E+07 | 2E+07 | 2E+07 |
| **0.1** | 800 | 96600 | 8200 | 0 | 2400 | 8000 | 2600 | 2000 | 13400 | 23400 | 4E+05 | 2E+06 |
| **1** | 1000 | 33200 | 2600 | 0 | 600 | 1400 | 1200 | 800 | 5200 | 5600 | ND | ND |
| **2** | 400 | 10400 | 2200 | 0 | 0 | 0 | 0 | 0 | 1600 | 1800 | 4E+05 | 2E+06 |
| **5** | 0 | 1200 | 200 | 0 | 0 | 0 | 0 | 0 | 1000 | 600 | 4E+05 | 1E+06 |
| **10** | 0 | 200 | 0 | 0 | 0 | 0 | 0 | 0 | 200 | 0 | 3E+05 | 4E+05 |
| **20** | 0 | 200 | 0 | 0 | 0 | 0 | 0 | 0 | 0 | 0 | 2E+05 | 1E+05 |
| **30** | 0 | 0 | 0 | 0 | 0 | 0 | 0 | 0 | 0 | 0 | 2E+05 | 4600 |
| **60** | ND | ND | ND | ND | ND | ND | ND | ND | ND | ND | 6800 | 0 |

| ***Escherichia coli*** | | |  |  |  |  |  |  |  |  |  |  |
| --- | --- | --- | --- | --- | --- | --- | --- | --- | --- | --- | --- | --- |
| **Time (min)** | **A** | **B** | **C** | **D** | **E** | **F** | **G** | **H** | **I** | **J** | **Cb** | **Lvx** |
| **0** | 4E+07 | 4E+07 | 4E+07 | 4E+07 | 4E+07 | 4E+07 | 4E+07 | 4E+07 | 4E+07 | 4E+07 | 4E+07 | 4E+07 |
| **0.1** | 2E+05 | 4E+05 | 2E+05 | 7600 | 2600 | 8800 | 2200 | 0 | 51400 | 200 | 4E+05 | 4E+05 |
| **1** | 82800 | 2E+05 | 2E+05 | 0 | 0 | 3200 | 0 | 0 | 800 | 0 | ND | ND |
| **2** | 44000 | 2E+05 | 38200 | 0 | 0 | 1200 | 0 | 0 | 0 | 0 | 4E+05 | 4E+05 |
| **5** | 4800 | 1E+05 | 1400 | 0 | 0 | 0 | 0 | 0 | 0 | 0 | 4E+05 | 79200 |
| **10** | 200 | 3000 | 400 | 0 | 0 | 0 | 0 | 0 | 0 | 0 | 3E+05 | 42200 |
| **20** | 0 | 0 | 0 | 0 | 0 | 0 | 0 | 0 | 0 | 0 | 2E+05 | 17400 |
| **30** | 0 | 0 | 0 | 0 | 0 | 0 | 0 | 0 | 0 | 0 | 2E+05 | 10200 |
| **60** | ND | ND | ND | ND | ND | ND | ND | ND | ND | ND | 6800 | 3400 |

Time (min)
